# Supplementary material for: Increased Pace of Aging in COVID-Related Mortality
Source: Life (Basel). 2021 Jul 22;11(8):730. doi: 10.3390/life11080730 (PMC8401657; doi:10.3390/life11080730)
Supplement: Supplementary file 1 [file life-11-00730-s001.zip › 23Jul2021_Supplementary_Materials.pdf]

Supplementary Materials

Metrics used

Metrics used to estimate BloodAge accuracy are Mean Absolute Error (MAE) and coefficient of determination (R^2), as defined in `sklearn.metrics`.

MedAE

$$MedAE = Median\left(\left|Age_{true,i} - Age_{predicted,i}\right|\right),$$
$$for\ all\ i\ \in (1, N), where\ N\ is\ the\ total\ number\ of\ samples$$

MAE

$$MAE = \frac{1}{N}\sum_{i=1}^{N}\left|Age_{true,i} - Age_{predicted,i}\right|, where\ N\ is\ the\ total\ number\ of\ samples$$

BloodAge prediction

BloodAge biological age measures can be obtained by uploading clinical blood tests to Aging.AI or Young.AI free of charge.

The models presented at these websites use adjustments not described in the original 2018 publication [1]:

$$BloodAge_{i,adj} = BloodAge_i - (BloodAge_{Mean}^{CA_i} - CA_i)$$

where: $BloodAge_i$ is the predicted age for the ith patient; CA_i is the chronological age of the ith patient.

$BloodAge_{Mean}^{CA_i}$ is the mean BloodAge for all people in the predictor's training data, whose chonological age is equal to CA_i.

This adjustment was used in the survival models used in this project. The full list of varaibles that can serve as input for BloodAge is as follows (in the order of descending importance): sex, BUN, RDW, CREA, MCV, ALB, GLC, NA+, ALP, CL, RBC, FERR, ALT, PLT, NEUTR%, MONO%, CHOLT, LDH, MCHC, TRIG, BILIT, EOS%, AST, LDL, HCT, HGB, GGT, K+, HDL, HGBA1C, CA, ESR, MCH, LYMPH%, BASO%, IRON, WBC, PROT, BILID, GLOBT, AAMY, UA, MPV, P, PDW, AFP, ATLYMPH.

Survival model data preprocessing

Methods

Training and verification data contained only the people infected with COVID. Patients with blood test discrepancies (immune cell counts not converging to WBC total count) were removed, as well as people with <30 blood parameters measured to ensure accurate BloodAge performance.

All the available blood biomarkers were transformed into two binary values to identify whether a patient's value is below or is above the leftover sample median. In case a missing value was encountered for a blood parameter, the median columns were both assigned zeroes. The model presented in the main text were trained using only the column that identifies if the patient belonged to the bottom half of the blood parameter distribution.

Smoking status was encoded as several alternative binary columns: "never smoker", "ever smoker", "current smoker". Body composition was encoded as two alternative binary columns: "overweight" (BMI>25 kg/m^2) and "obese" (BMI > 30 kg/m^2). The circulatory system state was encoded as one value — "HBP" (high blood pressure), which was equal to one if at admission both systolic pressure is above 140 mmHg and diastolic is above 90 mmHg. Otherwise, it was equal to zero. Sex was encoded as a value, that was equal to one if the stated sex was male and zero if it was female. Ethnicity was encoded as a binary value, which was equal to one if the person stated to be of African origin and zero otherwise. Biological age data was encoded as several alternative columns: delta age (BloodAge – actual age), underager (delta age < -3 years), overager (delta age > +3 years), aging group (-1 if the patient is an underager, +1 — overager, 0 — normal ager).

The blood parameters considered for inclusion into models were: whole blood immune cell count (WBC), ferritin (FERR), total protein (PROT), high density lipoprtotein (HDL), hemoglobin (HGB), mean corpuscular volume (MCV), alanine transferase (ALT), mean platelet volume (MPV), hematocrit (HCT), chloride (CL), serum albumin (ALB), total cholesterol (CHOLT), triglicerides (TRIG), direct bilirubin (BILID), total bilirubin (BILIT), sodium (NA+), red blood cell count (RBC), differential immune cells (BASO%, EOS%, LYMPH%, MONO%, NEUTR%), mean corpuscular hemoglobin concentration (MCHC), potassium (K+), aspartate transaminase (AST), fasting glucose (GLC), lactate dehydrogenase (LDH), total globulins (GLOBT), phosphorus (P), calcium (CA), low density lipoprotein (LDL), platelet count (PLT), glycated hemoglobin (HGBA1C), mean corpuscular hemoglobin (MCH), red blood cell distribution width (RDW), blood urea nitrogen (BUN), creatinine (CREA), alkaline phosphatase (ALP).

Medical history terms available for analyisis were: asthma, chronic obstructive pulmonary disease (COPD), coronary artery disease (CAD), congestive heart failure (CHF), chronic kidney disease (CKD), diabetes mellitus (DM), hypertension (HTN), cancer, number of comorbidities (N_history).

Admission symptoms available for analysis were: high blood pressure (HBP), fever chills, headache, dyspnea, gastro intestinal (GI), myalgia, cough, chest pain, altered mental state (AMS), number of symptoms (N_symptoms).

Final model feature selection

Methods

All survival models presented in this work were built using `Lifelines` library `v.0.23.9` for `Python 3`. To build Cox Proportional Hazards (CPH) `CoxPHFitter` method was used.

The optimal variables to be included in the final model were established via a grid search procedure. Alternative variables were defined; only one of them could be present in a single model.

CPH models were initialized with default settings. All models were trained with 5-fold cross-validation (CV) on a set comprising 75% of all the available settings. All models were verified on a set comprising 25% of all the available samples. The folds and the verification set remained the same across all models.

First, 17970 models were trained to select the 50 most predictive variables. In this stage the models contained no more than one blood parameter, as well as the alternative ways to define chronological age (continuous, binary with a threshold at 65 years, binary plus number of years over 65), biological age (delta age, aging group, under-/over-/normal ager columns, smoking (never smoker, ever smoker, current smoker), body composition (overweight, obese). Some models also included either the number of symptoms or comorbidities.

Each variable was rated according to the average c-index rank of the models it was a part of, 50 highest ranking variables were added into the second iteration. At this stage "never smoker" remained as the only smoking-related term in the top-50 list. A number of blood markers was removed at this stage.

During the second stage, 26100 models were trained using the 50 available variables. All the variables were scored in a similar fashion and 12 top-ranking variables were chosen for the final model, alongside "is male", "is black" and "never smoker" for a total of 15 variables in the final model.

C-index, or concordance index, is a metric of survival model performance. It was calculated using `concordance_index` from `lifelines.utils`. C-index is the proportion of sample pairs where the model guessed the last survivor right. Being a proportion, c-index fits within the unit radius. Random models produce c-index equal to 50%, perfectly accurate survival models have 100% c-index (or 0%, if all its predictions are reversed). All the models presented in this article used individual survival function expected value to guess the last survivor for the purpose of c-index calculation.

Results

A total of 26100 survival models were built for a CV subsample with 3987 patients, among which 1268 were confirmed dead at the end of observation (**Figure S1**).

The most descriptive features were selected as the 15 highest ranking variables (**Table S1**). Among them are five blood parameters: immune cell percentages (eosinophils, lymphocytes, monocytes, neutrophils), AMS, Dyspnea, DM, chronological age, delta age, BUN, LDH, creatinine, sex, race. All these variables, except age and delta age, are binary with cutoff values displayed in **Table S2**.

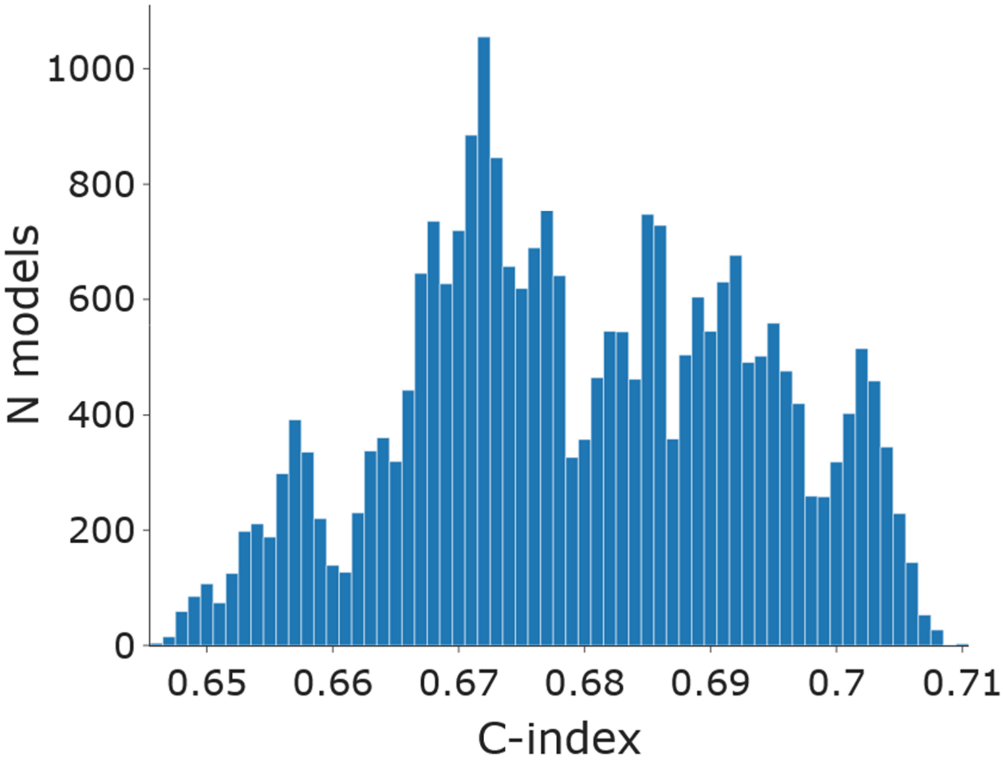

Figure S1 — Among the 26100 tested survival models, only 2498 show c-index of ≥0·70. C-index is the concordance index; c-index was calculated as the mean across five CV.

Survival classifier

<u>Methods</u>

Sensitivity and specificity in the survival status classifier are defined as:

$$Sensitivity = \frac{TP}{P} = \frac{TP}{TP + FN}$$

$$Specificity = \frac{TN}{N} = \frac{TN}{TN + FP}$$

where P is the total number of positive (dead at K days) patients; N is the total number of negative (alive after K days) patients; TP is the number of true positive predictions (predicted to die and is truly dead); TN is the number of true negative predictions (predicted to stay alive and is alive); FP is the number of false-positive predictions (predicted to die but was observed for longer); FN is the number of false-negative predictions (predicted to stay alive but was dead by the Kth day).

The best timeframe was established in terms of the least distance between sensitivity and specificity (the most “balanced” frame).

Results

The CPH classifier based on median time-to-death was used to calculate the sensitivity and the specificity in the verification set. The classifier shows its best performance when it is tasked with outcome prediction in 18 days — 62% specificity, 61% sensitivity.

While the verification set contains patients with extra long observation times, 90% of patients were observed for 24 days or less. In higher timeframes the sensitivity-specificity dependency is no longer monotonic, i.e. specificity may fluctuate at a constant sensitivity. This behavior is caused by the specifics of the metric calculation. People who were observed for T days and did not die would be eliminated from the metrics calculation in timeframes of >T days. If that person was predicted to survive for more than T days, this practically removes one true positive result which causes specificity (but not sensitivity) to noticeably decrease. The effects this shrinkage has on metrics are more noticeable in smaller samples — during longer timeframes when most patients are either no longer observed or dead.

Visualization

Data visualization is achieved with `Plotly` (`v.4.5.0`) for `Python` and `Seaborn (v.0.10.0).`

Table S1 — All the variables tested for inclusion into the final model (column "Included") sorted by the normalized average rank of the models they were a part of (column "Score"). Top-12 variables were added along with "Is male", "Is black", and "Never smoker", which were fixed at the previous stage of feature selection. "YrsOver65" was not included in the model, since it was ranked below "Age", which describes the same patient data dimension. "Is male", "Is black", and "Never smoker" were present in every model tested and thus had 0·50 score. The rows with "None" mark the models that used no information of the specified kind. Green marks the variables included in the final model

Variable	Score	Data type
Slice_bottom_MONO%	0.91	Blood_Marker
Slice_bottom_NEUTR%	0.90	Blood_Marker
Slice_bottom_LDH	0.86	Blood_Marker
Slice_bottom_CREA	0.81	Blood_Marker
Slice_bottom_LYMPH%	0.81	Blood_Marker
Slice_bottom_BUN	0.74	Blood_Marker
Slice_bottom_EOS%	0.64	Blood_Marker
Age	0.62	Age
Dyspnea	0.60	Symptoms
AMS	0.57	Symptoms
YrsOver65	0.54	Age
DM	0.53	History
Delta_age	0.52	BloodAge
Slice_bottom_GLC	0.52	Blood_Marker
Slice_bottom_K+	0.52	Blood_Marker
Slice_bottom_AST	0.52	Blood_Marker
COPD	0.51	History
CAD	0.50	History
GI	0.50	Symptoms
Is_overweight	0.50	Obesity
ASTHMA	0.50	History
HTN	0.50	History
Never_smoker	0.50	Smoking
Is_male	0.50	Sex
Is_black	0.50	Race
Is_obese	0.50	Weight
CKD	0.49	History
Aging_group	0.49	BloodAge
CANCER	0.49	Obesity
CHF	0.49	History
Underager	0.49	BloodAge
Overager	0.49	History
ChestPain	0.49	History
Headache	0.48	BloodAge
Cough	0.47	BloodAge
HBP	0.47	Symptoms
Myalgia	0.47	Symptoms
Slice_bottom_FERR	0.47	Symptoms
FeverChills	0.46	Symptoms
Over65	0.44	Symptoms
Slice_bottom_MPV	0.37	Blood_Marker
Slice_bottom_RDW	0.36	Symptoms
Slice_bottom_ALB	0.27	Blood_Marker
Slice_bottom_PLT	0.26	Blood_Marker
Slice_bottom_P	0.25	Blood_Marker
Slice_bottom_MCHC	0.23	Blood_Marker
Slice_bottom_CA	0.23	Blood_Marker
Slice_bottom_TRIG	0.22	Blood_Marker
history:None	0.16	Blood_Marker
blood_marker:None	0.15	Blood_Marker
symptoms:None	0.13	Blood_Marker

COVID-related mortality odds ratios

Methods

Odds ratio (OR) for binary variable *i* was defined as:

$$OR_i = \frac{a \div b}{c \div d} = \frac{N(dead|exposed\ to\ i) \div N(dead\ |unexposed\ to\ i)}{N(not\ dead\ |exposed\ to\ i) \div N(not\ dead\ |unexposed\ to\ i)}$$

$$\begin{aligned}
&where \\
&a\ is\ the\ number\ of\ lethal\ cases\ in\ which\ i = 1, \\
&b\ is\ the\ number\ of\ lethal\ cases\ in\ which\ i = 0, \\
&c\ is\ the\ number\ of\ non-lethal\ cases\ in\ which\ i = 1, \\
&d\ is\ the\ number\ of\ non-lethal\ cases\ in\ which\ i = 0
\end{aligned}$$

Confidence intervals (CI) for OR were calculated using the formula from [2]. Point estimate significance was estimated with chi2_contingency function from scipy.stats (v 1.4.1)for Python.

Results

See Table S2.

Table S2 — Odds ratios for all the binary present in the data.

	OR	2·5% CI OR	97·5% CI OR	Pval(Fisher)	Pval(Chi2)	Support in lethal cases (positive samples / non-event samples)	Support in non-lethal cases (positive samples / non-event samples)	Median cutoff (blood parameters only)
**Normal_ager**	1·03	1·17	0·91	6·57E-01	6·67E-01	1164/525	2476/1150	
**Over65**	3·12	3·51	2·77	8·15E-80	6·80E-80	1039/650	1229/2397	
**Overager**	0·92	1·07	0·79	2·66E-01	2·73E-01	289/1400	667/2959	
**Underager**	1·06	1·25	0·89	5·18E-01	5·46E-01	236/1453	483/3143	
**High ALB**	0·33	0·37	0·29	2·37E-76	6·62E-76	618/1071	2308/1318	35 g/L
**Low ALB**	3·16	3·57	2·81	9·65E-82	1·13E-81	1056/633	1252/2374	
**High ALP**	1·48	1·66	1·32	3·26E-11	3·89E-11	957/732	1700/1926	80 U/L
**Low ALP**	0·70	0·79	0·62	2·12E-09	2·26E-09	717/972	1860/1766	
**High ALT**	1·11	1·25	0·99	7·24E-02	7·58E-02	888/801	1810/1816	33 U/L
**Low ALT**	0·93	1·05	0·83	2·50E-01	2·53E-01	786/903	1750/1876	
**High AST**	2·22	2·50	1·97	1·26E-40	3·38E-40	1072/617	1591/2035	46 U/L
**Low AST**	0·47	0·52	0·41	4·19E-37	1·14E-36	601/1088	1967/1659	
**High BASO%**	0·55	0·62	0·49	3·32E-23	4·65E-23	676/1013	1981/1645	0·21 %
**Low BASO%**	1·81	2·03	1·61	2·51E-23	3·85E-23	1013/676	1644/1982	
**High BILID**	2·39	3·02	1·88	9·68E-15	1·84E-13	1600/89	3201/425	3·42 uM
**Low BILID**	0·42	0·54	0·32	1·42E-12	1·57E-11	74/1615	357/3269	
**High BILIT**	1·29	1·45	1·15	2·10E-05	2·34E-05	1031/658	1988/1638	8·55 uM
**Low BILIT**	0·81	0·91	0·72	3·35E-04	3·56E-04	643/1046	1570/2056	
**High BUN**	5·19	5·93	4·55	3·69E-149	2·45E-142	1318/371	1473/2153	6·43 mM

Low BUN	0·19	0·22	0·17	3·86E-147	4·05E-140	367/1322	2133/1493	
High CA	0·89	0·99	0·79	4·21E-02	4·34E-02	821/868	1872/1754	2·21 mM
Low CA	1·15	1·29	1·02	2·16E-02	2·31E-02	864/825	1732/1894	
High CHOLT	0·73	0·88	0·60	8·90E-04	1·13E-03	159/1530	454/3172	3·44 mM
Low CHOLT	1·25	1·49	1·05	1·42E-02	1·50E-02	220/1469	388/3238	
High CL	1·20	1·35	1·07	2·31E-03	2·53E-03	975/714	1931/1695	100 mM
Low CL	0·83	0·93	0·74	1·70E-03	1·79E-03	711/978	1694/1932	
High CREA	3·66	4·15	3·23	6·25E-100	1·78E-97	1203/486	1462/2164	84·9 uM
Low CREA	0·28	0·31	0·24	5·31E-98	1·50E-95	482/1207	2144/1482	
High EOS%	0·36	0·41	0·32	4·64E-63	4·64E-62	562/1127	2096/1530	0·19 %
Low EOS%	2·75	3·10	2·43	4·64E-63	4·64E-62	1127/562	1530/2096	
High FERR	1·93	2·17	1·71	7·32E-28	4·93E-28	839/850	1228/2398	1816 pM
Low FERR	0·49	0·56	0·44	2·24E-29	1·20E-28	472/1217	1593/2033	
High GLC	2·80	3·16	2·48	2·79E-65	3·26E-64	1132/557	1525/2101	7·05 mM
Low GLC	0·36	0·40	0·31	9·55E-66	1·09E-64	554/1135	2098/1528	
High GLOBT	1·36	1·55	1·20	3·69E-06	3·20E-06	502/1187	859/2767	32 g/L
Low GLOBT	1·03	1·18	0·89	7·42E-01	7·55E-01	347/1342	730/2896	
High HCT	0·79	0·89	0·70	6·94E-05	7·36E-05	788/901	1905/1721	38·3 %
Low HCT	1·27	1·42	1·13	6·94E-05	7·36E-05	901/788	1721/1905	
High HDL	0·78	0·94	0·65	9·89E-03	1·08E-02	175/1514	466/3160	0·83 mM
Low HDL	1·19	1·42	0·99	6·54E-02	6·98E-02	204/1485	376/3250	
High HGB	0·66	0·74	0·58	1·18E-12	1·42E-12	737/952	1962/1664	124 g/L
Low HGB	1·52	1·71	1·36	1·18E-12	1·42E-12	952/737	1664/1962	
High HGBA1C	1·09	1·27	0·93	3·01E-01	3·12E-01	290/1399	581/3045	6·6 %
Low HGBA1C	0·89	1·05	0·76	1·83E-01	1·92E-01	232/1457	549/3077	
High K+	1·67	1·88	1·49	5·33E-18	7·21E-18	991/698	1666/1960	4·3 mM

Low K+	0·57	0·64	0·51	4·29E-21	7·08E-21	649/1040	1895/1731	
High LDH	3·86	4·36	3·42	2·13E-110	1·78E-110	1073/616	1127/2499	441 U/L
Low LDH	0·25	0·28	0·22	5·81E-103	2·52E-97	343/1346	1838/1788	
High LDL	0·59	0·72	0·48	1·34E-07	3·31E-07	132/1557	456/3170	1·76 mM
Low LDL	1·34	1·60	1·12	1·84E-03	1·73E-03	219/1470	364/3262	
High LYMPH%	0·17	0·20	0·15	9·89E-171	9·63E-164	381/1308	2276/1350	13·26 %
Low LYMPH%	5·79	6·62	5·08	4·75E-171	5·78E-164	1308/381	1349/2277	
High MCH	1·06	1·19	0·94	3·31E-01	3·35E-01	872/817	1819/1807	28·9 pg
Low MCH	0·94	1·06	0·84	3·31E-01	3·35E-01	817/872	1807/1819	
High MCHC	0·49	0·56	0·44	2·86E-32	5·17E-32	665/1024	2058/1568	323 g/L
Low MCHC	2·02	2·27	1·80	2·86E-32	5·17E-32	1024/665	1568/2058	
High MCV	1·57	1·76	1·39	3·44E-14	4·39E-14	979/710	1697/1929	89 fL
Low MCV	0·64	0·72	0·57	3·44E-14	4·39E-14	710/979	1929/1697	
High MONO%	0·22	0·25	0·19	5·02E-133	7·97E-129	434/1255	2223/1403	6·27 %
Low MONO%	4·59	5·21	4·04	2·61E-133	5·07E-129	1255/434	1402/2224	
High MPV	1·71	1·92	1·52	2·08E-19	2·75E-19	1027/662	1724/1902	10·6 fL
Low MPV	0·51	0·58	0·45	1·16E-28	2·90E-28	575/1114	1822/1804	
High NA+	1·91	2·15	1·70	1·56E-27	3·12E-27	1047/642	1669/1957	139 mM
Low NA+	0·52	0·58	0·46	4·01E-28	6·54E-28	638/1051	1956/1670	
High NEUTR%	5·95	6·80	5·21	2·77E-175	9·03E-168	1315/374	1347/2279	77·75%
Low NEUTR%	0·17	0·19	0·15	5·85E-175	1·51E-167	374/1315	2278/1348	
High P	2·11	2·38	1·86	4·27E-32	8·61E-33	682/1007	882/2744	1·10 mM
Low P	0·96	1·09	0·84	5·16E-01	5·23E-01	480/1209	1063/2563	
High PLT	0·58	0·66	0·52	1·28E-19	1·65E-19	694/995	1974/1652	231x10E9/L
Low PLT	1·70	1·92	1·52	2·86E-19	3·43E-19	993/696	1652/1974	
High PROT	0·57	0·64	0·51	1·18E-21	1·37E-21	742/947	2103/1523	67 g/L

Low PROT	1·83	2·06	1·63	1·75E-24	1·88E-24	932/757	1457/2169	
High RBC	0·65	0·73	0·58	2·58E-13	3·14E-13	723/966	1943/1683	4·34x10E12/L
Low RBC	1·54	1·73	1·37	2·58E-13	3·14E-13	966/723	1683/1943	
High RDW	2·63	2·97	2·33	3·97E-58	2·52E-57	1116/573	1542/2084	13·8%
Low RDW	0·38	0·43	0·34	1·60E-58	1·27E-57	571/1118	2082/1544	
High TRIG	1·27	1·51	1·06	8·46E-03	8·87E-03	223/1466	388/3238	1·56 mM
Low TRIG	0·71	0·86	0·59	4·37E-04	5·57E-04	156/1533	454/3172	
High WBC	3·47	3·93	3·07	1·77E-92	2·44E-90	1192/497	1481/2145	63x10E9/L
Low WBC	0·29	0·33	0·25	1·77E-92	2·44E-90	497/1192	2145/1481	
AMS	2·66	3·06	2·31	3·17E-41	2·96E-43	495/1194	489/3137	
CAD	1·70	2·01	1·45	3·73E-10	1·88E-10	291/1398	395/3231	
HTN	1·67	1·89	1·48	1·55E-16	3·21E-16	1187/502	2124/1502	
CKD	1·63	2·00	1·33	3·70E-06	2·87E-06	175/1514	240/3386	
COPD	1·61	1·98	1·30	1·39E-05	9·93E-06	165/1524	229/3397	
CHF	1·48	1·79	1·23	4·88E-05	4·51E-05	203/1486	306/3320	
DM	1·44	1·62	1·28	6·24E-10	7·13E-10	875/814	1549/2077	
CANCER	1·08	1·30	0·90	4·42E-01	4·50E-01	186/1503	373/3253	
ASTHMA	0·88	1·05	0·75	1·60E-01	1·64E-01	218/1471	521/3105	
Ever_smoker	0·81	0·94	0·70	5·25E-03	5·59E-03	314/1375	796/2830	
Current_smoker	0·53	0·70	0·40	3·56E-06	7·65E-06	64/1625	252/3374	
Is_male	1·08	1·21	0·95	2·46E-01	2·50E-01	1088/601	2275/1351	
Is_obese	0·92	1·04	0·82	1·95E-01	2·03E-01	569/1120	1288/2338	
Is_overweight	0·90	1·01	0·79	8·08E-02	8·55E-02	1132/557	2517/1109	
Is_black	0·84	0·97	0·74	1·51E-02	1·60E-02	379/1310	926/2700	
Never_smoker	0·56	0·64	0·50	1·36E-21	9·89E-22	849/840	2326/1300	
Dyspnea	1·85	2·12	1·62	2·90E-20	1·32E-19	1318/371	2383/1243	

**HBP**	1·05	1·28	0·85	6·75E-01	7·03E-01	150/1539	309/3317	
**FeverChills**	0·81	0·91	0·72	5·18E-04	5·23E-04	933/756	2187/1439	
**Cough**	0·74	0·83	0·65	2·24E-07	2·46E-07	875/814	2153/1473	
**Myalgia**	0·65	0·77	0·55	7·16E-07	1·15E-06	199/1490	616/3010	
**ChestPain**	0·57	0·67	0·48	1·46E-11	4·79E-11	197/1492	686/2940	
**GI**	0·56	0·65	0·49	9·68E-17	3·22E-16	345/1344	1133/2493	
**Headache**	0·45	0·61	0·34	1·46E-08	6·24E-08	58/1631	264/3362	

Blood parameters in the COVID infected

Methods

All blood biomarkers had their distributions in lethal and non-lethal COVID cases approximated using Kernel Density Estimation (KDE). KDE was implemented using the `KernelDensity` method from `scikit-learn v0.22.1`. Kernel type was set to Gaussian, in all cases the bandwidth parameter was equal to 0.5 standard deviation of the feature within the total sample.

Results

We identified seven blood parameters as the most descriptive ones in terms of survival modeling. The cutoff values we used for some of them are outside the commonly used clinical reference ranges (**Table S3**). However, these reference ranges were derived for the healthy population and may be inappropriate while assessing the risks in the COVID-infected cohorts. Here we present the blood biomarker distributions in the COVID infected to illustrate that conventional definitions for "high" or "low" blood parameters are poor predictors of COVID-related mortality. Choosing the cutoffs that are within the normal range would result in an uneven proportion of the patients below and above the threshold (**Figure S2**).

Among the best blood predictors of COVID survival, all biomarkers show clear distinction between the distributions for lethal and non-lethal cases. In the cases of LDH, LYMPH%, MONO%, and NEUTR% the median cutoffs are located near the intersection of the lethal and non-lethal density functions, which is probably the reason for their superior discriminating properties.

For comparison, the non-predictive blood features, such as TRIG, have similar distributions within the lethal and non-lethal subsamples (**Figure S2**).

Table S3 — very few COVID patients have their blood parameters within the normal range, thus the cutoff values for binary variables were chose based on COVID sample medians. Green marks the blood parameters used in the final model.

Biomarker	Normal range [3]	N within, patients	N above, patients	N below, patients	N missing, patients
ALT	F:7-35; M:10-40 U/L	2919	2210	105	81
ALB	34-48 g/L	3212	34	1988	81
ALP	25-100 U/L	3701	1521	12	81
AST	10-30 U/L	1265	3955	11	84
BILIT	5-21 uM	4362	436	434	83
BILID	0-3.4 uM	431	4801	0	83
CA	2·15-2·5 mM	3230	199	1860	26
CL	98-106 mM	2774	890	1647	4
CHOLT	0-5·18 mM	1148	73	0	4094
CREA	F:53-97; M:62-115uM	2641	1757	893	24
FERR	F:22·5-270; M:45-562 pM	413	3709	10	1183
GLC	>60 years: 4·4-6·4 mM<br>≤60 years: 4·1-5·9 mM	1696	3454	159	6
GLOBT	22-40 g/L	2137	243	58	2877
HDL	F: >0·91; M: >0·75 mM	672	0	549	4094
LDH	208-378 U/L	1424	2751	206	934
LDL	<3·37 mM	1119	52	0	4144
P	0·87-1·45 mM	1841	639	627	2208
PROT	64-84 g/L	3467	106	1661	81
TRIG	<2·83 mM	1081	140	0	4094
BUN	2·1-7·1 mM	2712	2484	95	24
HCT	F:35-45; M:39-49%	2756	227	2332	0
HGB	F:120-160; M:135-175 g/L	2097	33	3185	0
WBC	4·5-11 x10E9/L	0	5315	0	0
MCH	26-34 pg	4573	66	676	0
MCHC	310-370 g/L	4249	10	1056	0
MCV	80-100 fL	4615	240	460	0
PLT	150-450 x10E9/L	4051	332	930	2
RBC	F: 3·8-5·1 x10E12/L<br>M: 4·3-5·7 x10E12/L	3059	253	2003	0
K+	3·5-5·51 mM	4608	260	333	114
NA+	136-146 mM	3352	696	1262	5
NEUTR%	57-67 %	745	4017	552	1
LYMPH%	23-33 %	717	383	4214	1
MONO%	3-7 %	2331	2264	719	1
EOS%	1-3 %	1006	340	3969	0
BASO%	0-0·75 %	5104	210	0	1

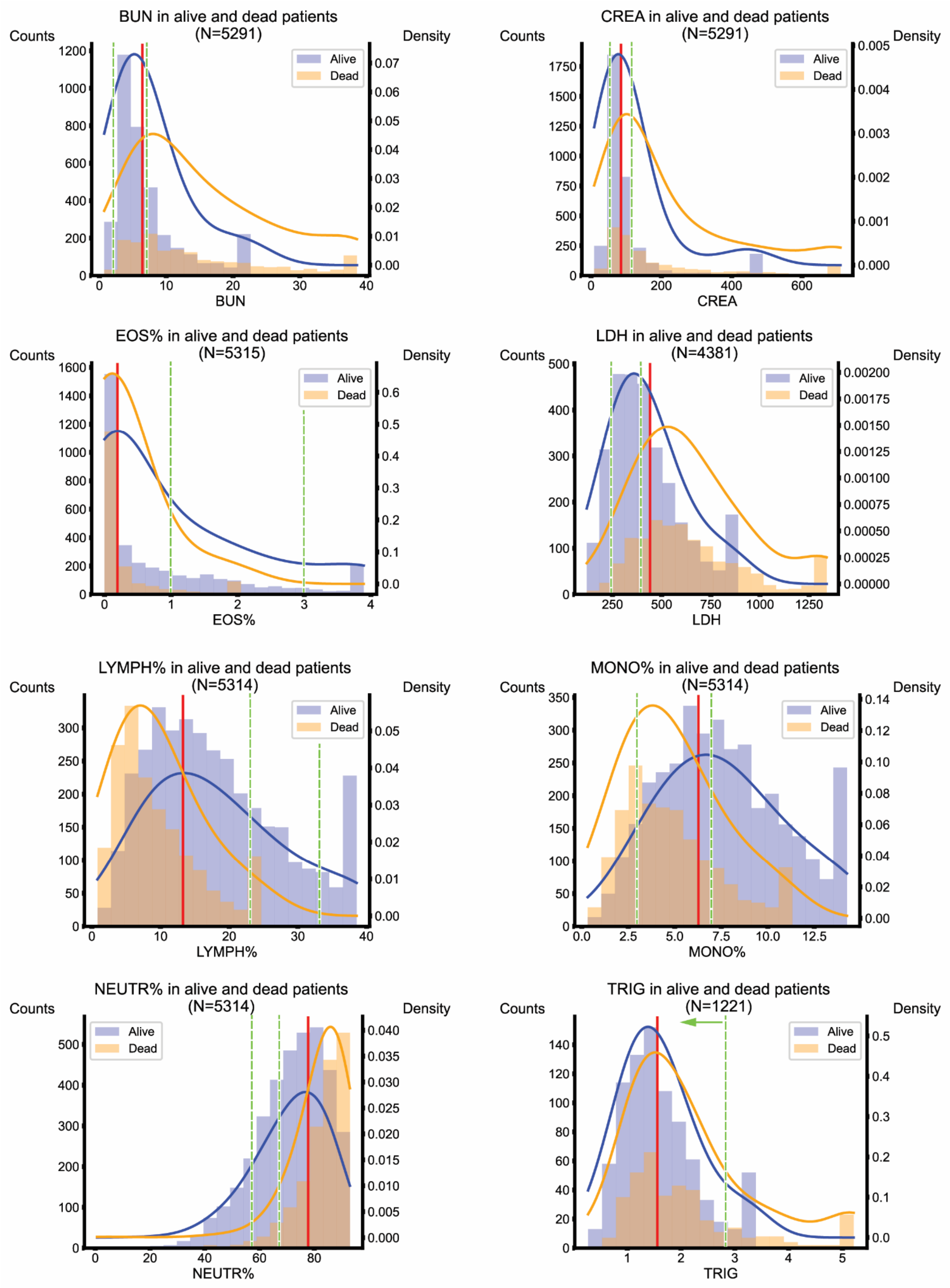
BUN in alive and dead patients
(N=5291)
Counts
Density
Alive
Dead
BUN
CREA in alive and dead patients
(N=5291)
Counts
Density
Alive
Dead
CREA
EOS% in alive and dead patients
(N=5315)
Counts
Density
Alive
Dead
EOS%
LDH in alive and dead patients
(N=4381)
Counts
Density
Alive
Dead
LDH
LYMPH% in alive and dead patients
(N=5314)
Counts
Density
Alive
Dead
LYMPH%
MONO% in alive and dead patients
(N=5314)
Counts
Density
Alive
Dead
MONO%
NEUTR% in alive and dead patients
(N=5314)
Counts
Density
Alive
Dead
NEUTR%
TRIG in alive and dead patients
(N=1221)
Counts
Density
Alive
Dead
TRIG

Figure S2. Distribution of blood parameter values in the total sample. BUN, CREA, EOS%, LDH, LYMPH%, MONO%, NEUTR% are used in the final model, while TRIG was identified as an insignificant blood biomarker (**Table S1**). N = Number of people within the total sample that had the corresponding blood biomarker measured. Blue and orange curves represent KDE estimates of the biomarker distributions (bandwidth = 0·3 × standard deviation). For display purposes, 5% of samples with the highest values were collapsed for into the histograms' last bin. Red lines mark the median cutoff values used to create binary variables. Green lines mark the reference ranges [3].

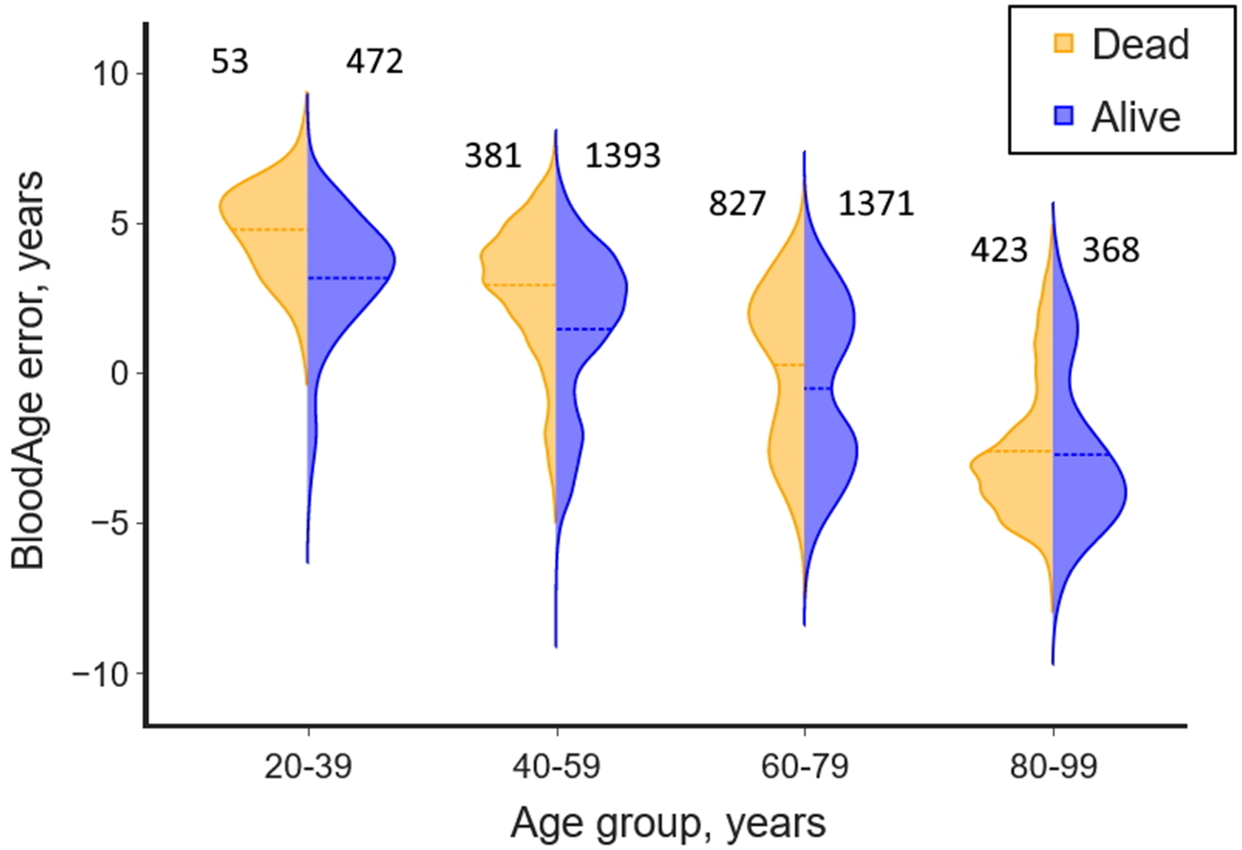

Figure S3 — Normalized violin plots for BloodAge error distributions. Prediction error depends on chronological age in the COVID sample. In all age groups, except for 80-99 years, the lethal cases had higher BloodAge errors. The number of patients in each subsample is marked above the violin. Dashed lines mark the mean values.

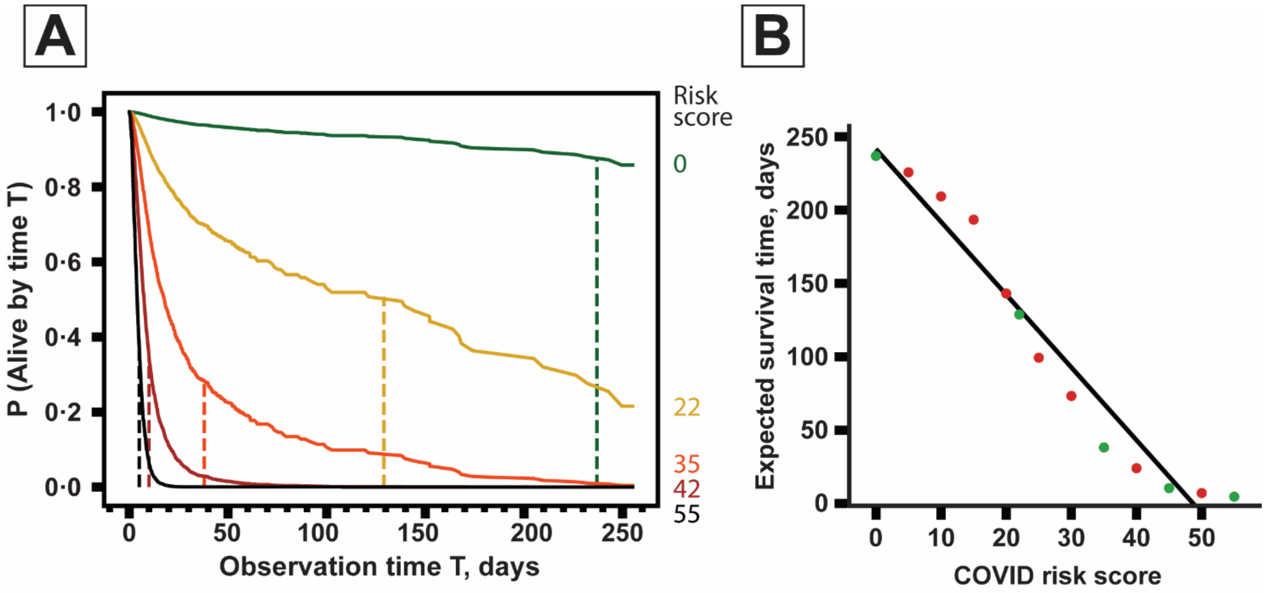

Figure S4 —

(A) Survival functions of four mock patients with COVID risk score assigned. The score belongs in the [0; 55] region, lower values indicate lower mortality risk and longer expected survival time (dashed lines).

(B) The proposed COVID risk score can be translated into the expected survival time using the formula obtained from linear regression: Time(days) = 242-5×Score. Green marks the points used as cutoff values for the COVID risk score.

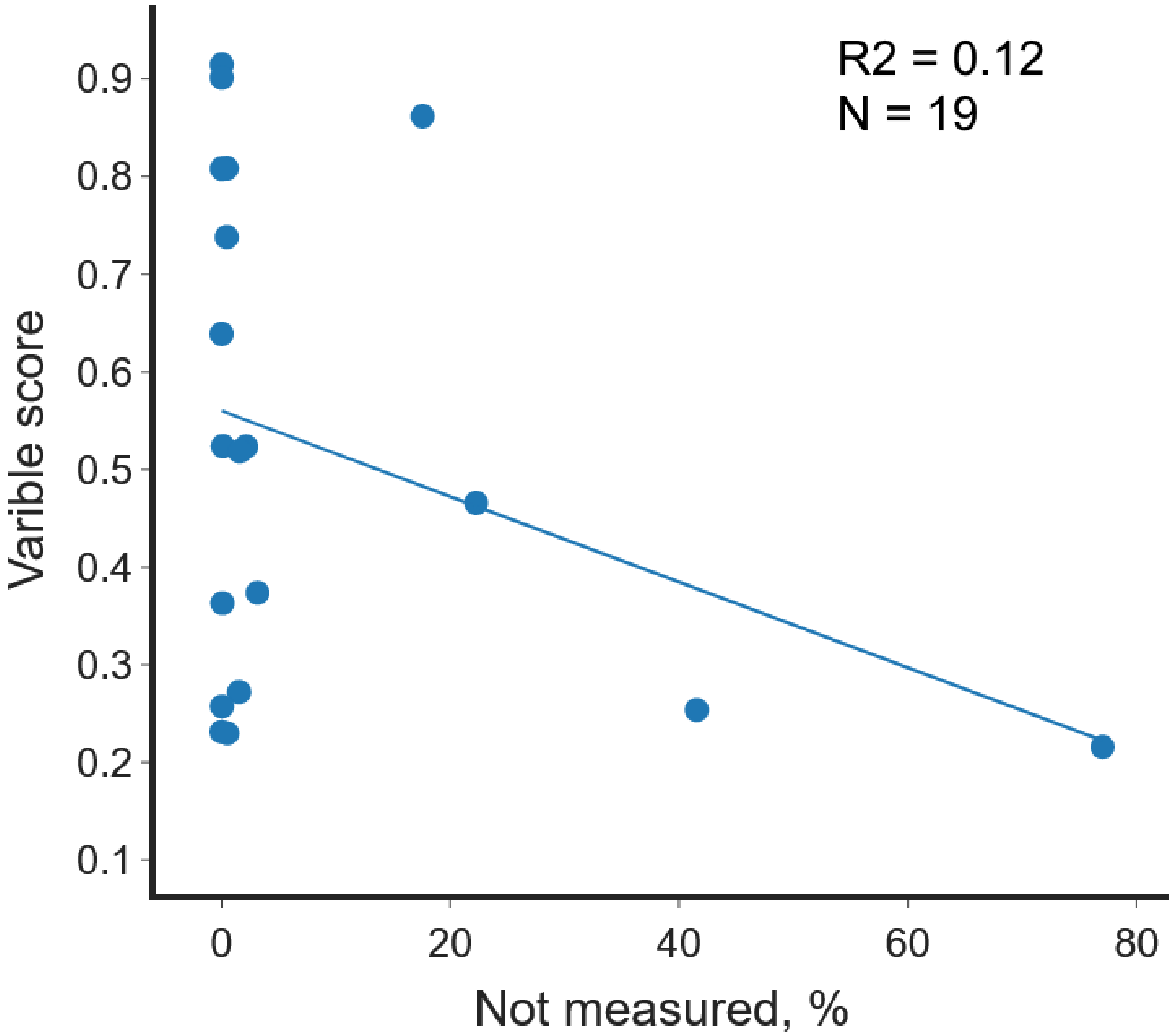

Figure S5 — The feature selection procedure eliminated most variables with a high number of missing measurements. The leftover features with a low number of missing values have a wide spectrum of importance scores.

R2 = Coefficient of determination; N = Total number of features plotted.

1 Mamoshina P, Kochetov K, Putin E, *et al.* Population Specific Biomarkers of Human Aging: A Big Data Study Using South Korean, Canadian, and Eastern European Patient Populations. *The Journals of Gerontology: Series A* 2018; **73**: 1482–90.

2 Szumilas M. Explaining Odds Ratios. *J Can Acad Child Adolesc Psychiatry* 2010; **19**: 227–9.

3 Goldman L, Plum F, Bennett CJ. Cecil Textbook of Medicine, 21st edn. 2000.
